# No axillary surgical treatment for lymph node-negative patients after ultra-sonography [NAUTILUS]: protocol of a prospective randomized clinical trial

**DOI:** 10.1186/s12885-022-09273-1

**Published:** 2022-02-20

**Authors:** Ji Gwang Jung, Se Hyun Ahn, Seeyoun Lee, Eun-Kyu Kim, Jai Min Ryu, Seho Park, Woosung Lim, Yong Sik Jung, Il Yong Chung, Joon Jeong, Ji Hyun Chang, Kyung Hwan Shin, Jung Min Chang, Woo Kyung Moon, Wonshik Han

**Affiliations:** 1 Department of Surgery, Seoul National University College of Medicine, 101 Daehak-ro, Jongno-gu, Seoul, 03080 Republic of Korea; 2 Department of Surgery, Asan Medical Center, University of Ulsan College of Medicine, Seoul, Republic of Korea; 3 Center for Breast Cancer, National Cancer Center, Goyang, Republic of Korea; 4 Department of Surgery, Seoul National University Bundang Hospital, Seoul National University College of Medicine, Seongnam, Republic of Korea; 5 Department of Surgery, Samsung Medical Center, Sungkyunkwan University School of Medicine, Seoul, Republic of Korea; 6 Department of Surgery, Yonsei University College of Medicine, Seoul, Republic of Korea; 7 Department of Surgery, Ewha Womans University School of Medicine, Ewha Womans University Mokdong Hospital, Seoul, Republic of Korea; 8 Department of Surgery, Ajou University School of Medicine, Suwon, Republic of Korea; 9 Department of Surgery, Gangnam Severance Hospital, Yonsei University College of Medicine, Seoul, Republic of Korea; 10 Department of Radiation Oncology, Seoul National University College of Medicine, Seoul, Republic of Korea; 11 Department of Radiology, Seoul National University College of Medicine, Seoul, Republic of Korea; 12 Biomedical Research Institute, Seoul National University Hospital, Seoul, Republic of Korea; 13 Cancer Research Institute, Seoul National University, 101 Daehak-ro, Jongno-gu, Seoul, 03080 Republic of Korea

**Keywords:** Breast cancer, Sentinel node biopsy, Ultrasound

## Abstract

**Background:**

Following sentinel lymph node biopsy (SLNB), the axillary recurrence rate is very low although SLNB has a false-negative rate of 5–10%. In the ACOSOG Z0011 trial, non-sentinel positive-lymph nodes were found in more than 20% of the axillary dissection group; the SLNB only group did not have a higher axillary recurrence rate. These findings raised questions about the direct therapeutic effect of the SLNB. SLNB has post-surgical complications including lymphedema. Considering advances in imaging modalities and adjuvant therapies, the role of SLNB in early breast cancer needs to be re-evaluated.

**Methods:**

The NAUTILUS trial is a prospective multicenter randomized controlled trial involving clinical stage T1–2 and N0 breast cancer patients receiving breast-conserving surgery. Axillary ultrasound is mandatory before surgery with predefined imaging criteria for inclusion. Ultrasound-guided core needle biopsy or needle aspiration of a suspicious node is allowed. Patients will be randomized (1:1) into the no-SLNB (test) and SLNB (control) groups. A total of 1734 patients are needed, considering a 5% non-inferiority margin, 5% significance level, 80% statistical power, and 10% dropout rate. All patients in the two groups will receive ipsilateral whole-breast radiation according to a predefined protocol. The primary endpoint of this trial is the 5-year invasive disease-free survival. The secondary endpoints are overall survival, distant metastasis-free survival, axillary recurrence rate, and quality of life of the patients.

**Discussion:**

This trial will provide important evidence on the oncological safety of the omission of SLNB for early breast cancer patients undergoing breast-conserving surgery and receiving whole-breast radiation, especially when the axillary lymph node is not suspicious during preoperative axillary ultrasound.

**Trial registration:**

ClinicalTrials.gov, NCT04303715. Registered on March 11, 2020.

## Background

Axillary surgery has been an essential breast cancer surgery since the introduction of Halsted radical mastectomy of the late nineteenth century. However, the survival benefit of axillary surgery has not been proven in a randomized controlled trial. In the National Surgical Adjuvant Breast and Bowel Project (NSABP) B-04 trial for clinically lymph node-negative breast cancer patients, overall survival (OS) was not different after radical mastectomy and total mastectomy without axilla surgery although approximately 40% of the radical mastectomy group were lymph node-positive [1].

Sentinel lymph node biopsy (SLNB) has replaced axillary lymph node dissection (ALND) for most cases of operable early breast cancer since it was introduced in 1994 to reduce the complication of ALND. In the NSABP B-32 study, the 8-year disease-free survival (DFS) did not differ in the ALND group and SLNB with ALND only group if the SLNs were positive (*p* = 0.54). The false-negative rate (FNR) for SLNB was 9.8% [2]. However, SLNB alone had significant complications. In the B-32 trial, the incidence of lymphedema was 7.5%, and the prevalence of sensory and other limitations of movement was 5–8%. Some studies have reported that SLNB only is associated with a reduced global health status QoL score [3].

The most important goal is to determine whether SLNB has a therapeutic role. SLNB is not a perfect procedure for removing metastatic lymph nodes. In the pivotal NSABP B-32 trial, the FNR of SLNB was 9.8% [2]. The FNR of the SLNB procedure was generally reported to be between 4.6 and 16.7% [4]. However, the axillary recurrence rates after SLNB only was between 0 and 1.5%, which is much lower considering the FNR of the SLNB procedure [5]. Furthermore, in the ACOSOG Z0011 study, 23.7% of the control group (axillary node dissection) patients had additional metastatic lymph nodes other than sentinel nodes. However, the axillary recurrence rate or DFS did not differ in the SLNB only and control groups [6]. These findings raise questions about the need to remove sentinel lymph nodes, especially in patients who undergo breast-conserving surgery and whole-breast radiation.

The axillary lymph node status became less important for deciding on adjuvant therapies for early breast cancer. In human epidermal growth factor receptor 2 (HER2)-positive or triple-negative breast cancers, adjuvant chemotherapy and/or HER2 targeted therapy was indicated regardless of lymph node status. For estrogen-receptor-positive HER2-negative breast cancers, multigene assays, such as the 21-gene or 70-gene assay, are used to guide adjuvant chemotherapy decisions.

With advances in imaging techniques, the accuracy of presurgical lymph node status prediction based on axillary ultrasonography (AUS) with or without core biopsy or fine-needle aspiration (FNA) has increased significantly. In a study by Cho et al., sonographic classification of axillary lymph nodes showed 85% sensitivity and 78% specificity for predicting metastasis when a cutoff for cortical thickness of 2.5 mm was used [7]. In the SOUND trial, preoperative AUS resulted in 15.5% of FNR, which was reduced to 8.1%, excluding isolated tumor cells and micrometastases, and 4.9%, considering only metastases of > 3 mm, which are the only lesions that AUS can reliably detect. The NPV was 95% after excluding isolated tumor cells and micrometastases [8].

Taking everything into consideration, this clinical trial aims to show whether SLNB can be omitted in a specific patient group. If there is no significant difference between the SLNB and non-SLNB groups, patients would have increased quality of life by eliminating unnecessary surgical procedures.

## Methods and design

### Study design

**Fig. 1:**
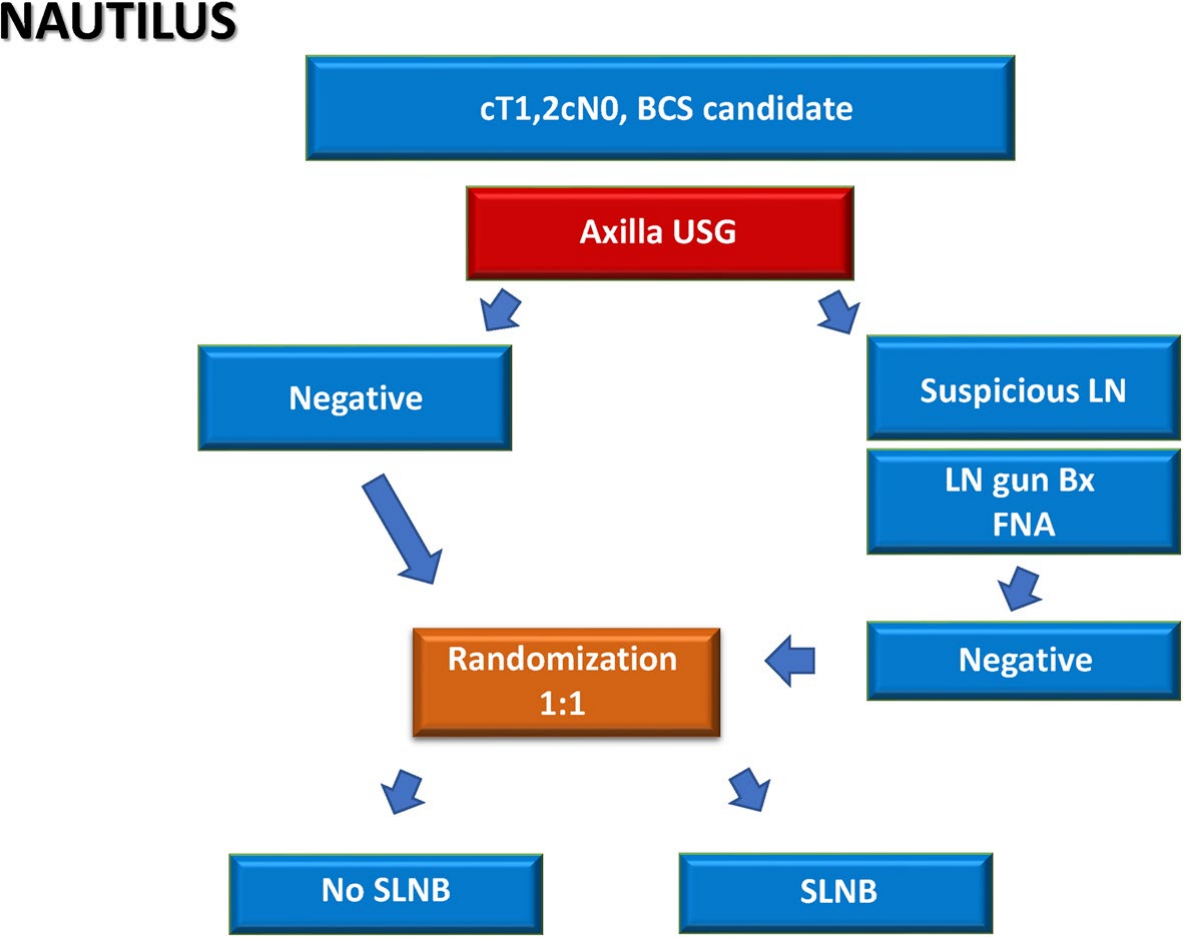
NAUTILUS trial: Study design

**Fig. 2:**
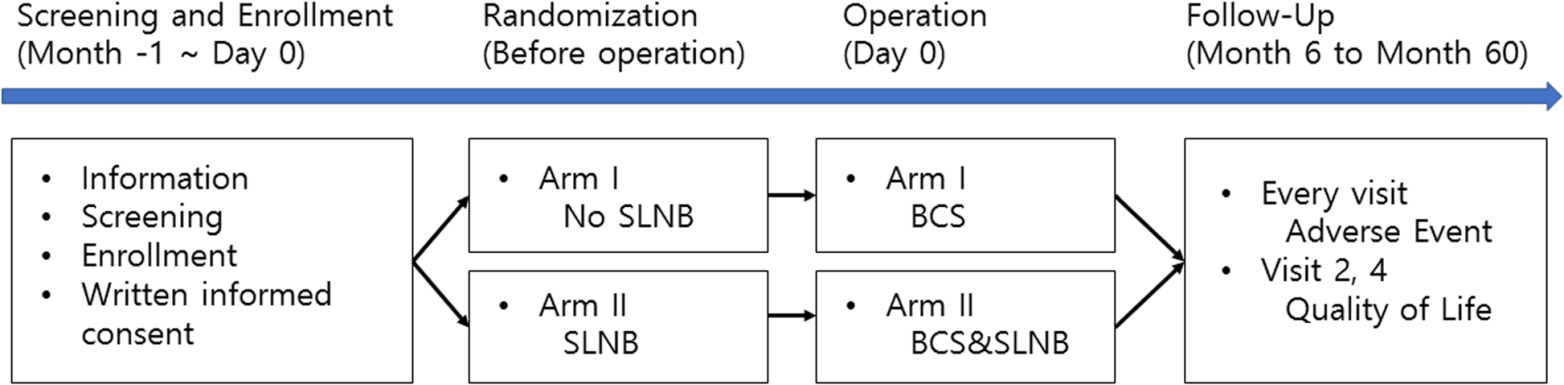
Diagram for the study process

The NAUTILUS trial is a prospective multicenter randomized controlled trial. Ten tertiary care hospitals in South Korea are participating in this trial. Patients with invasive breast cancer who have tumors less than 5 cm and clinically negative axillary lymph nodes and are expected to undergo breast-conserving surgery (BCS) are randomized (1:1) to the SLNB and no-SLNB groups. Eligible patients undergo AUS for the assessment of lymph node status before enrollment. Core-needle biopsy or fine-needle aspiration of the node is performed if a patient has a suspicious lymph node based on AUS findings (Fig. 1). The patients are blinded to the randomization process until the surgery. The study is expected to last for 7 years, with 2 years for registration and 5 years for follow-up (Fig. 2). Patient recruitment was started on September 1, 2020, and the first patient was recruited on September 15, 2020. The enrolment is estimated to be completed in April 2022.

The design and processes of the NAUTILUS study follow the guidelines for clinical trial protocols as specified by the Standard Protocol Items: Recommendations for Interventional Trials (SPIRIT) 2013 statement. This trial is registered at ClinicalTrials.gov (NCT04303715) on March 11, 2020.

### Study participants

Participating patients are included or excluded according to the specified criteria. The inclusion criteria are as follows: 1) 19 years or older with pathologically confirmed invasive breast cancer; 2) no clinical or radiological evidence of distant metastasis; 3) Eastern Cooperative Oncology Group (ECOG) performance status of 0–2; 4) negative axillary lymph node determined clinically and based on AUS; 5) for an eligible patient who has one minimally suspicious axillary lymph node based on AUS findings, confirmation of negative lymph node metastasis with core needle biopsy or FNA is mandatory; 5) BCS candidate with scheduled postoperative whole-breast irradiation; 6) follow-up after treatment is possible without physical, mental, or geographical restriction; 7) and full understanding of the written informed consent.

The exclusion criteria are as follows: 1) history of any cancer within the past 5 years except for well-treated skin cancers other than melanoma, thyroid cancer, and other in situ carcinoma excluding ductal carcinoma in situ of the breast; 2) bilateral breast cancer; 3) pre-treatment with neoadjuvant therapy; 4) pregnant or breast-feeding women; and 5) inability to understand and fill out questionnaires.

### Sample size calculation

We designed the NAUTILUS study to determine whether the no-SLNB group was not inferior to the standard SLNB group. In the NSABP B-32 study, the 5-year DFS was 88.6% for cT1–2 N0 breast cancer [2]. We assumed that the DFS of our study patients would be lower than this because lymph node-positive patients will be included in our study. In the ACOSOG Z0011 study, the 5-year DFS was 83.9% for cT1–2 N1 (1 or 2 lymph node-positive) [6]. We assumed that the DFS of our study patients would be higher than this. As a result, the expected 5-year DFS of the control group of our study is 86%.

The 5% non-inferiority margin, 80% power, 5% two-sided significance level resulted in a sample size of 780 per study arm. Assuming a 10% dropout rate, 1734 patients need to be randomized. Patients are recruited competitively from each institution.

### Screening and randomization

Participants undergo screening tests that will assess eligibility according to the inclusion/exclusion criteria. All procedures are performed after obtaining informed consent from the patients. Screening includes history taking, physical examination, pregnancy test, breast ultrasonography, and mammography. Additional tests can be performed if deemed necessary. AUS is performed by radiologists at each center to evaluate axillary lymph nodes. Written informed consent is obtained from each participant before enrollment by investigators. After enrollment, the randomization is conducted using a web-based system by investigators. Details of the randomization procedure will not be shared with the investigators of the clinical trial sites. The randomization table is prepared using SAS Version 9.4 (SAS Institute Inc., Cary, USA) with a stratified block randomization method. The stratification factors are treatment center and tumor size. The Medical Research Collaborating Center of Seoul National University Hospital (MRCC) created a random assignment table and operate it through web random assignment. Patients are blinded to their designated treatment arm before surgery to prevent bias or early dropout. During the randomization procedure, the investigator should provide the following information for each subject (protocol number, name of the clinical trial institution, screening number, tumor size). A randomization number is assigned through the web site, and a written confirmation of the subject is printed out and stored in the clinical trial basic document file provided to the institution. The randomization number applies to all case records and all subsequent correspondence related to the subject. Only pre-authorized investigators can access this randomization system.

### Interventions and follow up

Additional surgery involving the axilla after the results of SLNB are obtained in the control arm is dependent on the guideline of each treatment center. In both arms, adjuvant systemic therapy after surgery follows the guidelines of each center. Ipsilateral whole-breast radiation is mandatory for both arms. For the no-SLNB arm, the inclusion of axillary levels I and II within the tangential field or setting the upper margin of the field within 2 cm of the humeral head is recommended. The fractionation scheme follows 23–28 × 1.8–2.0 Gray (Gy) or 13–16 × 2.5–3.0 Gy. The boost to the tumor bed may be applied according to the policy of each center.

Follow-up of the patients will last for up to 5 years after surgery. The date of visit, status of disease progression, survival, adverse events, and quality of life findings will be reported at intervals of 3–6 months (according to each center’s policy). QoL questionnaires are acquired from the patients 1 and 2 years after the randomization using EORTC-BR23 and EORTC-QLQ-C30.

### Safety monitoring

In this study, we only gather adverse events related to axillary surgery according to the NCI/CTCAE v5.0 criteria followed by causality assessment. Severe adverse events should be reported to the primary investigator within 24 h of primary recognition of the event by the researcher. Severe adverse events should also be reported to the Institutional Review Board (IRB) within the timeframe designated by the IRB guidelines. Damages directly related with this trial to the subject that may occur during this clinical trial will be compensated in accordance with a clinical research agreement and insurance.

### Study outcomes

The primary endpoint is the 5-year invasive DFS, and the secondary endpoints are OS, distant metastasis-free survival, axillary recurrence rate, local recurrence rate, and self-reported adverse events. In addition, QoL will be evaluated twice at 1 year and 2 years after surgery. An interim analysis will be conducted after a 3-year follow-up.

### Data management and monitoring

Principal Investigator has full responsibility of entire projects of this trial. Clinical Research Organization (CRO) has roles of the coordinating center, CRO will help each hospitals which can proceed trial more effectively and will perform data monitoring and coordinate meetings of investigators. During patient recruitment monitoring on site is performed according to good clinical practice (GCP) guidelines. MRCC will perform data management team with data monitoring plan (DMP).

Data monitoring will be performed by CRO and clinical research coordinator (CRC) of each hospital and principal investigator will monitor entire data. In-house monitoring will be performed regularly with needs of investigators. Routine monitoring will be performed about 20 times with CRO and principal investigator.

All documents related to this clinical trial should be kept at the clinical trial institution. Subject (hospital or medical records) files are kept in accordance with domestic regulations. All documents related to this trial must be kept for 3 years after the end of the study. At the end of this period, the investigator obtains written approval from the trial coordinator before discarding the document. If a subject withdraws consent while participating in a clinical trial, data after the time of withdrawal of consent will not be collected. Research-related anonymized data and documents collected before the withdrawal of consent are retained and used for up to 3 years after the end of the study, and the investigator obtains written approval from the clinical trial coordinator of this study before discarding the documents when this period ends.

### Statistics

For nominal variables such as self-reported complications in each arm/group, the total number of patients, percentile, and frequency will be presented and analyzed using the chi-squared test or Fisher’s exact test. Continuous variables, such as age and tumor size, will be summarized using descriptive statistics (mean, standard deviation, median, etc.), followed by analysis using Student’s t-test or the Mann-Whitney U-test as a non-parametric test.

The statistical significance of the 5-year DFS, which is the primary endpoint, overall survival, and distant DFS will be determined using Kaplan-Meier analysis and the log-rank test. Cox proportional hazard regression analysis will be used to assess the prognostic significance of variables. For every statistical analysis, a *p*-value of < 0.05 will be considered statistically significant.

An interim analysis for the primary endpoint is planned once, and an interim analysis is performed 5 years from the date of IRB approval, and a final analysis is performed after at least 224 events have occurred. Disease-independent death is defined as a competing event. Fine and Gray regression is performed to verify the statistical hypothesis for comparison between groups, and the significance level used for interim and final analysis is calculated based on the O’Brien Fleming α-spending function, and the analysis uses SAS 9.4 Proc seq test.

## Discussion

The primary aim of the NAUTILUS study is to demonstrate the oncologic safety of omitting axillary surgery for selected lymph node-negative early breast cancer patients undergoing BCS and whole breast irradiation. The standard procedure for axillary surgery for these patients includes SLNB. Studies have reported that the 5-year axillary recurrence rate of patients with negative nodes detected by SLNB is 0.3–1.6% [5, 9–11]. The overall 8-year survival and 5-year DFS rates in the NSABP B-32 trial were reported to be 90.3 and 88.6%, respectively [2]. Considering that the FNR of the SLNB amounts to 5–10% and the SLNB does not completely get rid of metastatic lymph nodes, the aforementioned prognosis indicates that the unremoved metastasized lymph nodes do not lead to clinical recurrence in most cases. The very low axillary recurrence rate in the Z0011 trial also supports the hypothesis [6].

The morbidity rate after SLNB is generally lower than that after ALND; however, SLNB also has significant residual morbidity. In the NSABP B-32 study, arm volume differences of ≥10% at 36 months were evident for 8% of the SLNB group, and numbness and tingling peaked at 6 months for 15 and 10% of the SLNB group [12]. Kozak et al. reported undesirable sequelae, such as limited mobility in the shoulder joint, gradual increase in limb circumference, and pain, in patients after SLNB [13]. Based on the findings of other studies, lymphedema was reported in 7.5–9.0% of patients after SLNB after 6 months of follow-up and 5.2% even after 5 years of follow-up [12, 14–16]. Shoulder abduction deficit and sensory changes, such as numbness, pain, and paresthesia, have also been reported in patients after SLNB [12, 16, 17]. These morbidities are known to have physical, psychological, and emotional impact on the quality of life [18].

Eliminating or de-escalating axillary surgery aligns with the contemporary treatment trends. At least three clinical trials are being carried out, including the SOUND, INSEMA, and BOOG 2013–08 trials. The SOUND trial was designed to assess the non-inferiority of the 5-year distant DFS of cT1N0 breast cancer patients after SLNB to that after observation [19]. The INSEMA trial randomized cT1–2 N0 breast cancer patients into 4 groups, including the SLNB and SLNB-omission groups, and planned to analyze the 5-year DFS [20]. The BOOG 2013–08 trial also randomized cT1–2 N0 breast cancer patients into two groups, namely, the SLNB and no-SLNB groups, and evaluated the non-inferiority of regional recurrence in the no-SLNB group after 5- and 10-year follow-up [21]. These trials and our NAUTILUS trial altogether will answer the very important question that can alter the axillary surgery practice in breast cancer.

There are differences between this study and the aforementioned ongoing studies; however, this study has advantages. NAUTILUS is the only study targeting Asian breast cancer patients who are younger and more premenopausal than western women in terms of epidemiologic difference. Second, we have detailed ultrasonographic criteria for selecting patients and that is shared with breast radiologists (imaging) in each participating institution. Third, NAUTILUS aims to analyze the quality of life of each arm, which will add evidence to justify skipping SLNB. Cost-effectiveness will also be assessed for each arm.

In summary, the NAUTILUS trial aims to determine whether SLNB can be skipped for a specific breast cancer patient group. If there is no significant difference between the two randomized arms, patients who meet the criteria can have increased quality of life with less surgical morbidity and decreased medical costs but equivalent oncologic outcomes by avoiding unnecessary surgical procedures.

## Data Availability

Currently not applicable. Only the independant data monitoring commitee (IDMC) has access to unblinded outcome data before the trial ends.

## Abbreviations

ALND: Axillary lymph node dissection
AUS: Axillary ultrasonography
BCS: Breast-conserving surgery
FNR: False-negative rate
HER2: Human epidermal growth factor receptor 2
OS: Overall survival
QoL: Quality of life
SLNB: Sentinel lymph node biopsy
